# Diseases of Children

**Published:** 1915-03

**Authors:** E. Cecil Williams


### DISEASES OF CHILDREN.

**B. coli infections in infancy and childhood.**—The effects of the *B. coli* and its toxins on the organs affected and the symptoms resulting are so diverse and misleading, that a study of some of the more recent literature on the subject is of very great importance from the clinical standpoint. Hartshorne[1] quotes cases of invasion of the *central nervous system* in children by the *B. coli*, causing a meningitis clinically indistinguishable from other forms of meningitis. In these cases, mostly young babies, the colon bacillus has been grown in pure culture from the cerebro-spinal fluid. The condition was secondary to inflammation of the navel or bladder, otitis media, or spina bifida. Gordon[2] points out that in cases of coliuria in children the symptoms are not always associated with the urinary tract, but may simulate intracranial disease, especially meningitis, the onset being ushered in by convulsions, rigidity, and strabismus, and in some cases Kernig's sign may be present. I have recently had such a case under my own care, where meningitis was suspected, the cerebro-spinal fluid was clear and under considerable pressure, but contained no organism. A thorough examination of the urine, however, revealed coliuria, and when that condition was treated, all the other symptoms cleared up.

*Respiratory System.*—The *B. coli* is rarely the sole cause of inflammatory processes in the respiratory system. Pearson[3] reports a case of empyema, following bronchiectasis and pleurisy, in a boy aged seven years, where *B. coli* was found in pure culture in the discharge. In cases of coliuria the symptoms may suggest the onset of pneumonia, which, however, soon abate with the recognition of the urinary condition and the adoption of proper treatment.

*Intestinal Tract.*—The colon bacillus is always present in the intestinal tract, with the possible exceptions of (1) the condition known as the intestinal infantilism of Herter, and (2) for a few days in the newly born. It has not been proved that the colon bacillus, *per se*, is responsible for the many forms of intestinal disturbance in young children, though in cases of coliuria signs of abdominal disturbance, including vomiting, constipation, and sometimes diarrhœa, may be the only obvious symptoms present.

[1] *Med. Rec.*, 1914, lxxxvi. 288. [2] *Brit. J. Child. Dis.*, 1914, xi. 252.
[3] *Brit. M. J.*, 1909, ii. 78.

*Urinary Tract.*—These cases present far less difficulties in diagnosis, as the symptoms at once call attention to the affected system. The *B. coli* may be found in cases of enuresis, cystitis, pyelitis and pyelonephritis. The high acidity of the urine is usually responsible for the bladder symptoms, and there may be pain in the kidney region, usually the right. The mode of infection in these cases is not always the same; in girls it is usually an ascending infection, in boys transparietal; while in both sexes it may be by the blood.

All these various symptoms are doubtless the result of the circulation of toxins formed by the bacilli, which are in most cases neutralised by alkaline treatment, though alkaline treatment is not usually sufficient of itself to cause the disappearance of the bacilli from the urine. That such divergent symptoms should arise from the same cause only emphasises the necessity for thorough microscopical and bacteriological examination of the urine in all doubtful cases of illness in young children.

**Nervous Cretinism.**—McCarrison[1] describes this condition as a combination of infantile myxœdema (cretinism) with congenital cerebral diplegia in all their varying grades. Usually it is easy to distinguish the well-known signs of cretinism, but there are instances in which these signs are almost wholly wanting. The nervous symptoms may vary from the slightest degree of paraplegia to the most intense degree of spasticity, athetosis, fits and idiocy. Such extreme examples of this condition may be indistinguishable from cases of "cerebral diplegia," and it is only by the recognition of the scanty myxœdematous signs of the malady, and the application of the therapeutic test of thyroid medication, that their true nature can be appreciated. McCarrison is of opinion that the markedly beneficial result of thyroid medication is a clear indication that cases of this kind, which are not due to other obvious cause of cerebral diplegia, are often the results of thyro-parathyroid disease. The experimental work of Edmunds[2] shows that the effect of thyroidectomy in animals is such as to cause changes in the central nervous system, consisting of "chromatolysis of the cells large and small, swellings of the cell bodies swelling of the nuclei, extension of the nucleus, and total destruction of the cell body, leaving practically only a free nucleus. It is clear, therefore, that the effects of athyroidism being such, cases of thyroid deficiency should be recognised early, before permanent damage has been inflicted on the neurones. The amount of benefit derived will depend on the duration of the disease prior to the commencement of the treatment.

[1] *Proc. Roy. Soc. Med.* (Sect. Dis. of Child.), 1914, vii. 157.

[2] *Tr. Path. Soc. Lond.*, 1902, liii. 343. *Proc. Roy. Soc. Med.*, (Neur. Sect.), 1912, v. 179.

**Status Lymphaticus.**—The extreme importance of the recognition of this disorder cannot be too strongly insisted upon, as it so often leads to unexpected and untoward results. The most constant feature is enlargement of the thymus, associated with more or less general lymphoid hypertrophy all over the body. The normal area of thymic dulness is V-shaped, and does not transgress the margin of the manubrium sterni, while in enlargement of the gland this area passes from half an inch to three-quarters of an inch to either side of the bone and below joins the cardiac dullness. If with these physical signs there is a tendency to unexplained attacks of dyspnœa, or to a persistently low vitality, as evidenced by subnormal temperatures and intolerance of exertion, the "status lymphaticus" should be suspected, and the child should not be unnecessarily exposed to the risk of an anæsthetic. Thursfield[1] is of opinion, from a consideration of recent experimental work on the thymus, that the "status lymphaticus" is not merely due to disorder of the thymus, but to a much more complicated disorder of the whole ductless gland system. He advises prior to the administration of an anæsthetic, in all cases where this condition may be suspected, the injection of pituitrin, an extract which has the most extraordinary effect in warding off shock.

E. Cecil Williams.

### Reviews of Books.

[1] *Proc. Roy. Soc. Med.* (Sect. Dis. of Child.), 1914, vii. 186.

